# “I know what I'm supposed to do, but I don't do it”: patient-perceived risk factors that lead to their lower extremity amputations

**DOI:** 10.1186/s13047-023-00675-3

**Published:** 2023-11-13

**Authors:** Marcelle Ben chmo, Lisa Matricciani, Saravana Kumar, Kristin Graham

**Affiliations:** 1 Allied Health & Human Performance, The University of South Australia, North Terrace, Adelaide, SA 5000 Australia; 2 Clinical and Health Sciences, The University of South Australia, North Terrace, Adelaide, SA 5000 Australia; 3 Innovation, IMPlementation and Clinical Translation (IIMPACT in Health), The University of South Australia, North Terrace, Adelaide, SA 5000 Australia

**Keywords:** Type 2 diabetes mellitus, Lower extremity amputations, Qualitative descriptive

## Abstract

**Background:**

The purpose of this study is to extend on our previous research by exploring patient-perceived factors that lead to their Lower Extremity Amputations (LEA). LEA are a serious complication of Type 2 Diabetes Mellitus (T2DM), LEA are thought to be preventable with early detection and management of risk factors. Our previous study identified that these factors extend beyond the typical biological and modifiable risk factors and may also extend to patient awareness and competing priorities. Therefore, this research explored these issues in further detail, identifying patient-perceived factors that lead to their LEA.

**Methods:**

A qualitative descriptive methodology involving non-probability purposive sampling was used to recruit inpatients at a tertiary metropolitan hospital in South Australia. Semi-structured interviews were conducted, and data were transcribed verbatim. Data from the interviews were analysed using thematic analysis and the constant comparison approach.

**Results:**

A total of 15 participants shared their perspectives of risk factors for LEA. Two main themes emerged: intrinsic and extrinsic factors. Intrinsic factors identified in this study included identity, ambivalence, denial, inevitability, and helplessness. Extrinsic factors related to resources, rapport with healthcare professionals, and management of care.

**Conclusions:**

Through identifying that a combination of perceived personal attributes (intrinsic) and system-level (extrinsic) factors likely contribute to LEA, this study highlights the complexity of factors that contribute to patients' perceptions of what led to their diabetes related LEA. These findings support the importance of a nuanced approach in managing patients with diabetes who are at risk of LEA as it's likely patients’ personal circumstances, day-to-day life’s requirements and responsibilities, their interaction with healthcare professionals all seemingly contribute to how risks are viewed and managed. Tackling this challenge will require reimagining diabetes care, acknowledgement of risk factors beyond the obvious and addressing persistent access and workforce issues.

**Supplementary Information:**

The online version contains supplementary material available at 10.1186/s13047-023-00675-3.

## Background

Lower Extremity Amputations (LEA) are a serious and costly complication of Type 2 Diabetes Mellitus (T2DM) with a five-year mortality rate of 30.5% [1]. While complications of diabetes have been associated with reduced quality of life, the largest decrements in self-reported quality of life in T2DM occur in people who experienced a LEA [2]. Similarly, the prevalence of depressive symptoms and cardiovascular comorbidities have been found to be higher in T2DM patients with LEA, compared to those without a LEA. Therefore, LEA are a considerable burden to both the individual and the health system [3]. In Australia, diabetes-related LEA are estimated to cost the healthcare system $48 million per year, with the total cost per amputation being $23,555 and an additional expenditure of $6,065 every year afterwards [3, 4]. Efforts to prevent diabetes-related LEA are therefore an important health priority [3, 4].

Current diabetes-related LEA prevention strategies tend to focus on early detection and management of biological and modifiable risk factors [4, 5]. These risk factors include peripheral neuropathy, peripheral vascular disease, cigarette smoking, poor glycaemic control, structural foot deformities, and infection [4, 5]. The multifactorial nature of complications leading to LEA, a multidisciplinary approach is often recommended and has been demonstrated to reduce amputation rates by 39–56% [6]. Indeed, approximately 85% of diabetes-related LEA are thought to be preventable with appropriate primary prevention and specialist care [7].

In Australia, rates of diabetes-related LEA are higher than the global average [8, 9]. These findings are somewhat surprising given that Australia has a universal health insurance scheme (Medicare) which guarantees all Australians access to a wide range of health services at low or no cost [10]. Those with chronic diseases such as diabetes are eligible to access a care plan through Medicare which provides heavily subsidised access to allied health services. Given the availability of free and subsidised services, it is of interest and importance to better understand why potentially preventable LEA continue to be a growing problem in Australia.

Studies of patient perceptions have provided unique and valuable insights in many areas of health including the impact of diabetes treatment on quality of life [11]. While there are studies that examine the lived experience of patients with a LEA, we are unaware of any qualitative studies, except of our own recent study [12], which explored patient perspectives for the development of a LEA as a result of T2DM [12–15]. Our study identified themes relating to patient awareness and competing priorities as important risk factors that confound a patient’s ability to manage their risk of LEA [12]. These factors extend beyond the typical biological and modifiable risk factors and reflect the complexities that underpin what patients' encounter in their everyday lives [12]. However, it is still unknown what are patient-perceived factors that lead to the foot complications in the first place [12].

The aim of this study was to address this knowledge gap by exploring patient-perceived factors that lead to their LEA. This is of relevance to South Australia, where this study was undertaken as South Australia has one of the nation’s highest prevalence rates of LEA as a result of diabetes foot complications [8, 16].

## Methods

### Research design

This study applied a qualitative descriptive (QD) research design to explore patient perceptions of factors that lead to their LEA [17]. QD methodology has previously been used in other qualitative research to gain perspectives of population groups about cultural and clinical factors [18–21].

The conduct and reporting of this research were informed by the consolidated criteria for reporting qualitative research [22].

### Participants

Patients admitted to a tertiary metropolitan hospital in South Australia due to a diabetes-related LEA were invited to take part. Participants with cognitive impairment or who were not fluent in verbal and written English were excluded. Senior podiatrists at the tertiary South Australian metropolitan hospital screened participants for eligibility, provided patients with an information sheet, and gained verbal consent from the patient to be contacted by the principal researcher. The principal researcher then formally invited the patient to take part in the study and both written and verbal informed consent was obtained. This process aimed to avoid any coercion to participate and ensure the decision to participate was independent of the treating health care professionals. Participants were informed that participation was voluntary, that all data would be de-identified, and that they could withdraw from the study at any time without impacting their treatment.

A non-probability purposive sampling method was used to recruit participants over a two-month period (November–December 2020). This sampling method is suited to studies that have a predefined sample of interest so that a particular phenomenon can be examined. In this study, participants who have experienced a LEA because of T2DM. A sample size of approximately 12—15 participants was estimated to be suitable based on comparable research, feasibility, time, and resources which is comparable to similar studies in this field [23, 24].

### Data collection

Face-to-face semi-structured interviews with open-ended questions were conducted by the principal researcher, with an average length of 45 minutes. Interviews provide an opportunity to gain a meaningful, insightful, and deep understandings of participant’s experiences [24–26]. The semi-structured style allowed the interviewer to request additional information for greater exploration of responses. Twenty-one interview questions focused on the patient’s perceptions of factors contributing to LEA as a result of complications from the diabetes foot (Additional File 1). The interview guide was developed by the research team following a review of the literature and consultation with practising podiatrists with a special interest in the diabetes foot. The interview protocol was piloted with the research team and provided the principal researcher with interview practice. All interviews were audio recorded and written field notes were recorded to provide context to each interview.

### Data analysis

Data from the semi-structured interviews were transcribed verbatim by an independent transcription company. A small sample (20%) were independently transcribed verbatim by the principal researcher, who is a female podiatrist. Data were managed using the QSR-NVivo software TM (Version 12). Thematic analysis was conducted using the constant comparison approach [17, 22, 27], as this allows rich descriptive data to be summarised while still maintaining its depth [27]. A subset of transcripts was double coded by KG, a female academic who has tertiary qualifications in podiatry and psychology, a PhD, and experience and training in qualitative analysis, and LM a female academic with tertiary qualifications in podiatry and nursing, a PhD, and experience and training in qualitative analysis, to ensure methodological rigor and consistency. The principal researcher completed coding the remaining transcripts. Emerging themes were discussed and refined with an experienced qualitative researcher (SK), an academic with tertiary qualifications in physiotherapist and extensive qualitative research experience, throughout the analysis process for clarity and rigour. Themes were developed through repeated reading of and immersion in each transcript [24]. Frequent meetings during the coding process provided the opportunity to discuss interpretations of the transcript and compare codes and themes to ensure similar ideas were identified, and to resolve coding discrepancies through discussions. Relevant quotations were selected to illustrate main points within each theme.

Trustworthiness of data analysis was addressed through strategies to optimise credibility, dependability, transferability, and confirmability [28]. Interview questions were piloted by the principal researcher to ensure adequate coverage of the issues of interest. To ensure the data collection was credible and dependable, the principal researcher undertook interview training prior to collecting data [24]. Further, to minimise any influence of interpretation or bias by the researcher all interviews were conducted with participants who had no personal nor professional relationship with the principal researcher. The principal supervisor (KG) attended the first three interviews as an independent observer and to provide the opportunity for peer debriefing. Field notes were used as a means of cross-checking findings and audiotaped to ensure credibility of the data [27]. Multiple coders for some interviews, peer debriefing, adherence to a semi-structured interview guide, and transcribing verbatim by an external transcription company also promoted triangulation, credibility and confirmability [24, 26].

## Results

Eighteen in-patients admitted for a diabetes-related foot complications and a history of a LEA agreed to participate in the research project. Of the 18 participants, three were excluded due to a family member or a partner being present during the interview and spoke for the participant. This meant the data collected did not truly reflect the patient’s perspectives. A final sample of 15 participants were examined in this study. As presented in Table 1, most (86%) participants were male and Caucasian, with a median age of 66.4 years ranging from 44–80 years. The median duration of diabetes was 25.2 years, ranging from 12–40 years. More than half of the participants had undergone a previous amputation with 86% being low-income earners or retired and 73% living in metropolitan Adelaide.

**Table 1:** Participant characteristics

Participant ID	Gender	Age (years)	Ethnicity	Working status	Residential Location in SA^a^	Duration of T2DM (Years)	Number of LEA
P1	Female	53	Caucasian	Employed	Metropolitan	12	1
P2	Male	78	Caucasian	Retired	Metropolitan	40	2
P3	Male	59	Caucasian	Retired	Metropolitan	20	2
P4	Male	80	Caucasian	Retired	Metropolitan	15	2
P5	Female	44	Caucasian	Unemployed	Metropolitan	26	1
P6	Male	65	Caucasian	Retired	Metropolitan	15	1
P7	Male	57	Caucasian	Employed	Metropolitan	32	2
P8	Male	76	Caucasian	Retired	Rural	32	1
P9	Male	65	Caucasian	Retired	Rural	25	2
P10	Male	82	Caucasian	Retired	Metropolitan	20	1
P11	Male	63	Caucasian	Retired	Rural	35	1
P12	Male	72	Aboriginal	Retired	Rural	40	2
P13	Male	72	Caucasian	Retired	Metropolitan	30	1
P14	Male	71	Aboriginal	Retired	Metropolitan	10	1
P15	Male	59	Caucasian	Retired	Metropolitan	26	2

^a^Rural was defined as any residential location included in the Australian bureau of section of state, category two (bounded locality) and three (rural balance) [22]

Analysis of the data identified two overarching themes, intrinsic and extrinsic factors each with associated subthemes (Table 2). Intrinsic factors related to how participants perceived their diabetes and lower extremity amputation, from their individual perspective. The subthemes for intrinsic factors included identity, ambivalence, denial, inevitability, and helplessness. Extrinsic factors related to a participant’s environment and external influences impacting their own health. The subthemes for extrinsic factors were resources, management of care, and rapport with healthcare professionals. The results below are organised around these themes and subthemes.

**Table 2:** Themes, and subthemes of patient’s perceptions of risk factors that contributed to their LEA

Themes	Sub themes	Summary
Intrinsic factors	Identity	Elements of identity impacted participants perceptions of their risk factors e.g., occupational, father, rural, independent, self-image, health
	Ambivalence	Having good knowledge of the risk factors but acting to the contrary
		Not managing their risk factors due to complacency
	Denial	Participants perceived they had good health despite negative outcomes such as amputation
	Inevitability	Perceptions of the certainty of the outcome resulted in lack of engagement in risk management
	Helplessness	Participants believed they were unable to control or change the outcome
Extrinsic factors	Resources	Difficulties with accessing healthcare e.g., transport, lack of appointments and choice of healthcare professional
	Rapport with healthcare professionals	Negative perceptions about communication, trust, and support from healthcare professionals
	Management of care	The perception that negative outcomes were due to poor healthcare management

### Theme 1: intrinsic factors

#### Sub theme 1: identity

Identity of the individual was a factor that contributed to diabetes related LEA. One participant described occupational identity in terms of protecting themselves against diabetes related complications:

*P15: ‘…there's a certain amount of image involved as well. If you get a guy like me, hard drinking, ex-army, down at the pub with all your mates "I'm tough, I can handle it." You never think of a bacteria bringing you down.’*

Another participant expressed how occupational identity influenced how he managed his chronic disease:

*P13: ‘I’ve always worked as a FIFO miner, it’s hard to look after diabetes or feet when you’re in that environment.’*

Identifying with the role of a father was also described. One participant reported how they wanted to hide their diabetes as they did not want to burden others. In doing so, they neglected diabetes self-management:

*P10: ‘I didn’t tell anyone I had diabetes because I care a lot about my family and I didn’t want them to worry, I’m the father and I should look after them instead of them looking after me… I was careful about what I done at home and would eat things I shouldn’t or not check my sugars so no one knew.’*

Participants also identified as being from a rural area and discussed this identity as a factor that led to their LEA:

*P11: ‘I'm barefooted, I'm- I'm a country boy…when those ulcers started, I could of taken it a bit more seriously but I’m from the country so I thought I’d be fine.’*

This was supported by another participant who reflected that *P8: ‘In the bush I would just keep working and smoking, didn’t really worry about my health.’*

The need to maintain a social identity of independence impacted some participants seeking help for diabetes foot complications:

*P12: ‘I ignored it because I still want to be independent, I don’t want people to worry about me. I didn’t look after my diabetes because I was too stubborn and wanted to do things on my own.’*

Maintaining a self-image of being healthy impacted how participants perceived risk from their diabetes. One participant stated:

*P2: ‘It’s just about maintaining that image, even when people tell you about your health, you still think you can handle it. You still think you can do it. I can handle this. Other people can't, but I can.’*

On the other hand, in some instances, participants prioritised others health over their own:

*P2: ‘I always thought that there’s other people who would need it more than me, so I tried to not bother anyone with my diabetes.’*

#### Sub theme 2: ambivalence

Ambivalence related to participants having the awareness of and knowledge about their diabetes, understanding the negative consequences of their actions, and yet acting to the contrary. For example, one participant reported that they knew they should be wearing better footwear, however because of the inconvenience chose not to adhere:

*P11: ‘I probably could wear better footwear. At the moment I’m just wearing sandals because it’s easier and I can’t be bothered.’*

Some participants perceived they didn’t need to change their current behaviours:

*P2: ‘They (healthcare professionals) tried to get me to wear shoes when I had the first amputation, but I thought no I can walk, and I thought there's no need for it.’*

Such sentiments were supported by another participant who reflected:

*P10: ‘I know what I'm supposed to do, but I don't do it…I stopped seeing the dietitian, I've more or less stopped seeing the diabetes educator as well. Once they tell you what you have to know, again, complacency and I don't need to be told anymore.’*

#### Sub theme 3: denial

Denial related to participants state of denial about their own health, which ranged from rationalising to ignoring to complete denial. One participant perceived they had good health outcomes despite having an amputation and accepted no responsibility for that outcome:

*P11: ‘I think my diabetes and my health is really good except for this amputation which isn’t my fault anyway.’*

For some, denial seemingly was as a simpler option as reflected by this participant:

*P4: ‘I just ignored the wound. Put a couple of band aids on it and that was it. That's all, just easier.’*

Other participants were able to identify and articulate they were in a state of denial about managing their diabetes. For one participant this was despite having good knowledge and education about diabetes:

*P3: ‘I was in the state of denial that I had type 2 diabetes and the silly thing is I actually lectured and tutored medical students and science students. In diabetes, both type 1 and type 2. So, I knew it very well, even at the molecular level. I completely ignored the symptoms …just blocked it out or ignored it.’*

*P5: ‘It's just, I stuck my head in the sand, just hope it disappeared. I’ve had that kind of stuff explained, like the numbness and how to look after it and everything else. I just didn't prioritize it.’*

For one participant, who had diabetes for more than two decades, it was about rationalising what occurs when living with diabetes.

*P9: ‘I mean, I've been diabetic for 25 years and you do hear it, but you don't necessarily think about your foot like the first time that happens. You might take some notice of that and then …bad outcomes …because I'll find a way to rationalize what I've been told to work for me.’*

In some instances, participants believed that such consequences (such as LEA) would not occur to them.

*P13: ‘I thought that an amputation and even diabetes would happen to other people, not me.’*

#### Sub theme 4: inevitability

Inevitability related participants perception that diabetes and its consequences were inevitable and therefore unavoidable.

*P4: ‘Every single member of my family has got diabetes, every single one. And they all died because of it…so I'm next in line.’*

For some participants the perception of inevitability lead to a lack of proactive behaviour in managing their personal risks:

*P5: ‘I knew about diabetes back when I was 33 when it killed my brother. I still didn't learn anything about me, didn’t have myself checked out and that's where I went wrong. If I'd had myself checked out, then I could've, I could've avoided a lot of problems.’*

The feeling of inevitability resulted in one participant feeling disempowered and impacted their motivation to engage in self-management strategies:

*P10: ‘Since I had the amputation, I don’t think there’s anything I’ll change and there’s nothing I can do differently… it is what it is. I haven’t seen my podiatrist in a while… I’ve only got one foot they can do now since the amputation, and I’ll probably lose the other one anyway.’*

#### Sub theme 5: helplessness

Building on from inevitability, helplessness related to participants’ experience of a sense of helplessness when confronted with diabetes:

*P14: ‘Can't do it. I can't do anything for my diabetes. All I can do is sit at home and wait for the care mob to come and just do it.’*

For one participant having had a LEA resulted in a belief that nothing else could be done:

*P12: ‘I won’t be no different, my legs are gone now so you know, they might only see me once in a blue moon or whatever.’*

The potential for a negative outcome from diabetes seemingly further fostered feeling of helplessness:

*P1: ‘They (healthcare professional) told me about smoking that so bad, it's going to kill me. I knew about lungs and all that but not my diabetes or that it will kill me but I was like, Oh, god I don't care. I can't cope anymore.’*

*P11: ‘I mean what else could they do as far as my toe… nothing it was going to be cut off anyway.’*

Other participants felt that their personal past experiences with diabetes lead to inevitable outcomes that couldn’t be altered as it was too late:

*P2: ‘You start to realize that you need to manage your diabetes and look after yourself by the time you start losing your limbs, but then it's too late.’*

### Theme 2: extrinsic factors

#### Sub theme 1: resources

Resources were identified as an extrinsic factor influencing access to health care which in turn contributed to LEA. Transport to attend healthcare appointments was an obstacle for participants from both rural and metropolitan areas.

*P14: ‘I can’t see anyone else for my diabetes because I don’t have any transport*’.

For some participants, difficulties with transport limited the variety of healthcare professionals that they could access.

*P12: ‘I was joined up with the podiatrist through the Country Health, but I've got no vehicle, and I cannot access a vehicle to go there. We have no support to get anywhere so I only see the GP (General Practitioner) now for my diabetes’.*

Two participants reported that outcomes from their diabetes including neuropathy and a previous amputation impacted their ability to drive and they had difficulty sourcing other means of transport.

*P11: ‘It’s hard getting to appointments… I don’t have any transport. I’m not allowed to drive because I can’t feel the pedals with my diabetes.’*

*P4: ‘It’s hard to get to appointments when you have to rely on other people, with the leg off I haven’t driven for about two years now.’*

Timely availability of appointments for healthcare consultation was an issue for those living in rural areas. Staff shortages limited not only access but also choice.

*P8: ‘It's really hard in the bush…you don't get the appointments… you’ve got the need and we don't have the people with the qualifications to see the needs. In the bush you have to be satisfied with whatever doctor you get, there’s not any choice.’*

#### Sub theme 2: rapport with healthcare professionals

Rapport with healthcare professionals related to several facets of service delivery and its impact on participants perception of their diabetes related outcomes. These included communication, trust, and support. The following quote illustrates a participant’s concern that the healthcare professional did not care about his diabetes.

*P9: ‘More or less, he (GP) didn't give a mug about my diabetes.'*

Some participants perceived that healthcare professionals were aloof and did not provide adequate explanation.

*P12: ‘The GP didn’t explain to me how diabetes affects your feet, he’s quite detached. But I’m not sure how to go to another doctor.’*

While others perceived healthcare professionals as being procedural and impersonal.

P6: ‘They basically sit down and ask you 10 questions, tick, cross, tick, tick, cross. Sign the sign list…you do get the general impression that give a sh*t anyway.’

For one participant, poor communication resulted in feeling unsupported, negatively affecting engagement in their own healthcare.

*P5: ‘I have issues with the diabetes clinic. It wasn't a good fit, like personality clashes …I didn't feel supported…I had a diabetic nurse come in and talk to me, like I was the worst piece of rubbish, and was going off at me, but she wouldn't even listen to, you know, these are the circumstances. She just kept going and going and going at me. I just, I shut down. I just literally just shut down and thought, think what you want. I just shut down.’*

Another participant however provided a different view. They expressed that because the onus for the negative consequences wasn’t placed on them, they perceived that the healthcare professional didn’t care.

*P1: ‘I must admit I was very disgusted with a professional's attitude to what had happened to my feet. It didn't seem to matter much to them… I was never made to feel guilty as in, you wouldn't be like this if you hadn't, if you'd followed the rules.’*

In some instances, the context in which the healthcare professional worked seemed to influence this. One participant expressed the difficulty in building rapport with healthcare professionals who were not based in their local area.

*P9: ‘People are also very trust oriented in the country (rural area) and there’s no healthcare professionals that want to stay and live in the country so I’m also very hesitant to see anyone.’*

#### Sub theme 3: management of care

Management of care related to participants perceptions of variations in how their diabetes care was delivered potentially contributing to negative outcomes. This is highlighted by the following quotes by participants who stated:

*P1: ‘When I went to hospital that time I fell over, nobody ever bothered to check the bottom of my feet and so I ended up with a fairly large ulcer on the bottoms of both feet.’*

*P7: ‘I needed an amputation because I had an infection, and the infection wasn’t stopped by the doctors or nurses.’*

Similarly, one participant perceived that not a single healthcare professional had provided adequate care for their diabetes:

*P9: ‘I went to six doctors, four podiatrists and two specialists… yet no one did anything about my diabetes.’*

Another participant provided an explanation for this. They expressed that due to the long duration of their diabetes there was a lack of education provided from their podiatrist.

*P2: ‘The podiatrist didn’t really talk to me about diabetes and how this affects my feet but its most likely because I had diabetes for 30 years before I started seeing her.’*

## Discussion

Through exploring patient-perceived factors that lead to their LEA, this study identified a myriad of intrinsic and extrinsic factors. These findings suggest that a combination of perceived personal attributes (intrinsic) and system-level (extrinsic) factors likely contribute to LEA. By identifying these factors, this study has highlighted the importance of a nuanced approach in managing patients with diabetes who are at risk of LEA. This is essential as it’s likely patients’ personal circumstances, day-to-day requirements and their interaction with healthcare professionals all seemingly contribute to how risks are viewed and managed.

### Intrinsic factors

In this study, perceived identity was an intrinsic factor that influenced an individual’s perception of their risk factors. Forms of identity related to occupation, fatherhood, ruralness, and independence. Some participants perceived their occupational and rural identity was associated with a ‘toughness’ or a lifestyle that made self-management difficult. While those identifying with fatherhood and social identity of wanting to maintain independence either did not want to burden others or wanted to ‘do things on their own’. A developed framework identifies five corresponding abilities (ability to perceive, seek, reach, pay and engage) that individuals’ need to access to health care, highlighting the importance of social determinants of health [29]. A primary healthcare approach that understands these determinants of health and patient-perceived factors may help healthcare professionals tailor education and self-management strategies to meet patient’s needs. Such as recognising, that the way a patient sees themselves may impact an individual’s ability to perceive, seek, reach, and engage in primary level healthcare [29].

Other factors including ambivalence, denial, inevitability, and helplessness reflected barriers that contributed to participants LEA. These barriers were often found to be inter-related. For example, some participants were in denial about their own health, rationalising they had a good outcome despite an amputation. Some participants ignored their condition, while others felt helpless and as though complications and poor health outcomes were inevitable. The theme of ambivalence reflected that despite understanding the possible negative outcome of a LEA participants acted on the contrary, by denying any immediate risk. Ambivalent participants often denied the chronic nature of their diabetes and perceived outcomes were inevitable, so again they felt helpless. Corscadden et al. 2017 highlights that barriers to healthcare occur as a result of intrinsic factors which can form a chain reaction effect and is only as strong as its weakest link [30]. Demonstrating that these intrinsic factors don’t occur in isolation but rather co-exist and may influence one another. Identifying this link between factors may further help individuals achieve the ability to perceive, seek, reach, pay and engage in health care [29].

Our findings of denial, inevitability, and helplessness support previous findings that this powerful emotion can result in difficulty in engaging with self-care and adherence to treatment particularly in chronic diseases [31]. It has been suggested that the diagnosis of a chronic illness can result in a reaction that resembles the stages of mourning, of which denial is a fundamental step, and that moving onto acceptance involves building a new identity [32]. Patience and understanding is needed to provide a safe and trusting environment to encouraging patients experiencing denial to develop a sense of autonomy in managing their diabetes [31, 33].

### Extrinsic factors

One extrinsic factor uncovered centred around resources, for example participants found it difficult to access healthcare due to a lack of transport, appointments, and choice of healthcare professional. Further, the lack of transport compounded the ability to attend healthcare appointments and reduced the choice of healthcare professionals that participants could access. This issue was even more amplified for those in rural and remote areas. Previous studies have reported that poor access to healthcare services increased the risk of LEA [33]. For example, people in rural areas have a higher incidence of LEA compared to those who live in metropolitan areas [34]. In South Australia, rates in rural areas of Ceduna and Flinders are 37.9–111.7 people per 100,000 compared to Port Adelaide rates of 22.3 people per 100,000 [35]. Our findings suggest that outcomes in diabetes foot disease may be improved through access to transport, particularly in rural areas, and increased choice of healthcare professionals. This is supported by Levesque, Harris and Russell 2013 who highlight that a key strategy to better help manage chronic diseases includes access to healthcare and service delivery [28]. Staff shortages in healthcare, particularly in rural areas, is a long-standing issue. However, alternative methods of accessing healthcare, such as telephone consults and telehealth have shown promise in improving access to both generalized and specialist medicine appointments particularly for rural and disadvantaged groups [36, 37].

An important finding of this research was that participants had negative perceptions about communication, trust, and support from healthcare professionals, which impacted acceptability and rapport and therefore the ability to engage. This was amplified when care was provided by a locum healthcare professional who was only visiting for a short period of time. Patient-provider communication is an important factor in treatment adherence. Non-adherence to treatment is found to be higher in patients' who report poor patient-provider communication [38]. Further, participants had the perception that their negative health outcomes were likely due to poor healthcare management. Participants perceived a lack of intervention, ‘no one did anything’, as well as an inadequate provision of education. Our findings support previous research which identified that poor patient satisfaction and the perception that they do not receive adequate information are significant factors in treatment non-adherence [39–41].

Many system level strategies have been developed to address improved patient engagement and outcomes. Patient-centred care and increasing patient involvement in healthcare innovation have become a national priority and yet in practice, most interventions are still designed without the input of the patients they are intended to benefit [40, 42]. While the current staff shortages and priority in dealing with emergent health crisis (such as the COVID-19 pandemic) may be contributing to this policy-practice gap, improved understanding of patient’s perceptions of the factors that lead to LEA can help closing this gap. For T2DM related LEA to decline, the status quo requires challenging. This will likely require reimagining diabetes care which brings together biological, psychological, social, and cultural factors as well as addressing persistent access and workforce issues.

While this research has contributed to the knowledge base on patient-perceived factors that lead to their LEA, it is not without limitations. As a qualitative study conducted within a single South Australian tertiary hospital setting, the transferability of the findings is limited. However, the findings do provide first-hand perceptions of patients admitted to hospital for a LEA. To build on this body of research, future studies in other hospital and health settings may be beneficial as well as examining healthcare professional’s perception of factors that influence LEA.

## Conclusions

Despite a reduction in T2DM, Australia and particularly South Australia has an increasing number of LEA. The findings from this research indicate that myriad of factors underpin patients' perceptions of their LEA. From our previous study we know that these factors go beyond the typical biological and modifiable risk factors to include psychological, social, and cultural factors^13.^ The current research builds on these previous findings by highlighting the complexity of factors that patients perceive as leading to their LEA. This is a combination of perceived personal attributes (intrinsic) and system-level (extrinsic) factors. Tackling this challenge will require healthcare professionals to reimagine diabetes care, acknowledgement of risk factors beyond the obvious and addressing persistent access and workforce issues.

### Supplementary Information

**Additional file 1.** Interview guide 

## Data Availability

The datasets used and/or analysed during the current study are available from the corresponding author on reasonable request.

## Abbreviations

CALHN HREC: Central Adelaide Local Health Network Human Research Ethics Committee
COREQ: Consolidated Criteria for Reporting Qualitative Research
LEA: Lower Extremity Amputations
QD: Qualitative Descriptive
T2DM: Type 2 Diabetes Mellitus
UniSA: University of South Australia

## References

[1] Jupiter DC, Thorud JC, Buckley CJ, Shibuya N (2016). The impact of foot ulceration and amputation on mortality in diabetic patients. I: from ulceration to death, a systematic review. Int Wound J.

[2] Hayes A, Arima H, Woodward M (2016). Changes in quality of life associated with complications of diabetes: results from the ADVANCE study. Value in Health.

[3] O’Neill SM, Kabir Z, McNamara G, Buckley CM (2017). Comorbid depression and risk of lower extremity amputation in people with diabetes: systematic review and meta-analysis. BMJ Open Diabetes Res Care.

[4] Lin C, Liu J, Sun H (2020). Risk factors for lower extremity amputation in patients with diabetic foot ulcers: a meta-analysis. PLoS One.

[5] Adelman RD, Tmanova LL, Delgado D, Dion S, Lachs MS (2014). Caregiver burden: a clinical review. JAMA.

[6] Albright RH, Manohar NB, Murillo JF (2020). Effectiveness of multidisciplinary care teams in reducing major amputation rate in adults with diabetes: a systematic review & meta-analysis. Diabetes Res Clin Pract.

[7] Buckley CM, Ali F, Roberts GA, Kearney PM, Perry IJ, Bradley CP (2015). Timing of access to secondary healthcare services and lower extremity amputations in patients with diabetes: a case–control study. BMJ Open Diabetes Res Care.

[8] Dillon MP, Fortington LV, Akram M, Erbas B, Kohler F (2017). Geographic variation of the incidence rate of lower limb amputation in Australia from 2007–12. PLoS One.

[9] Narres M, Kvitkina T, Claessen H (2017). Incidence of lower extremity amputations in the diabetic compared with the non-diabetic population: a systematic review. PLoS One.

[10] Services Australia. What health care is covered by Medicare, how to enrol and how to claim. Australian Government. https://www.servicesaustralia.gov.au/medicare. Accessed 20 Jan 2023.

[11] Bradley C, Speight J (2002). Patient perceptions of diabetes and diabetes therapy: assessing quality of life. Diabetes Metab Res Rev.

[12] Ben Chmo M, Matricciani L, Kumar S, Graham K (2022). “I was trying to look after myself, but I really wasn’t”: understanding patient’s perspectives on risk factors for lower extremity amputations. J Foot Ankle Res.

[13] Ong EKM, Fryer C, Graham K, Causby RS (2022). Investigating the experience of receiving podiatry care in a tertiary care hospital clinic for people with diabetes related foot ulcers. J Foot Ankle Res.

[14] Zhu X, Goh LJ, Chew E, Lee M, Bartlam B, Dong L. Struggling for normality: experiences of patients with diabetic lower extremity amputations and post-amputation wounds in primary care. Prim Health Care Res Dev. 2020;21. 10.1017/S146342362000064X. 10.1017/S146342362000064X PMC7801928 33323161

[15] Coffey L, Mahon C, Gallagher P (2019). Perceptions and experiences of diabetic foot ulceration and foot care in people with diabetes: a qualitative meta-synthesis. Int Wound J.

[16] Narres M, Kvitkina T, Claessen H (2017). Incidence of lower extremity amputations in the diabetic compared with the non-diabetic population: a systematic review. PLoS One.

[17] Sandelowski M (2000). Whatever happened to qualitative description?. Res Nurs Health.

[18] Kim H, Sefcik JS, Bradway C (2017). Characteristics of qualitative descriptive studies: a systematic review. Res Nurs Health.

[19] Graham K, Banwell HA, Causby RS, Kumar S, Tian EJ, Nissen L (2021). Barriers to and facilitators of endorsement for scheduled medicines in podiatry: a qualitative descriptive study. J Foot Ankle Res.

[20] Thorpe O, Kumar S, Johnston K (2014). Barriers to and enablers of physical activity in patients with COPD following a hospital admission: a qualitative study. J Chron Obstruct Pulmon Dis.

[21] Dankiw KA, Kumar S, Baldock KL, Tsiros MD (2023). Parent and early childhood educator perspectives of unstructured nature play for young children: a qualitative descriptive study. PLoS One.

[22] Tong A, Sainsbury P, Craig J (2007). Consolidated criteria for reporting qualitative research (COREQ): a 32-item checklist for interviews and focus groups. Int J Qual Health Care.

[23] Feinglass J, Shively VP, Martin GJ (2012). How ‘preventable’are lower extremity amputations? A qualitative study of patient perceptions of precipitating factors. Disabil Rehabil.

[24] Patton, MQ. Enhancing the quality and credibility of qualitative analysis. *Health Serv Res.*1999;34(5 Pt 2):1189–208. PMC1089059 10591279

[25] Lewis S (2015). Qualitative inquiry and research design: Choosing among five approaches. Health Promot Pract.

[26] Hurworth R (2006). Research design in social research; successful qualitative health research: a practical introduction. Eval J Australas.

[27] Liamputtong P, Ezzy D (2013). Qualitative research methods.

[28] Guba EG, Lincoln YS (1994). Competing paradigms in qualitative research. Handb Qual Res.

[29] Levesque J-F, Harris MF, Russell G (2013). Patient-centred access to health care: conceptualising access at the interface of health systems and populations. Int J Equity Health.

[30] Corscadden L, Levesque JF, Lewis V (2017). Barriers to accessing primary health care: comparing Australian experiences internationally. Aust J PrimHealth.

[31] Silva JAd, Souza ECFd, Echazú Böschemeier AG, Costa CCMd, Bezerra HS, Feitosa EELC (2018). Diagnosis of diabetes mellitus and living with a chronic condition: participatory study. BMC Public Health.

[32] Rinnenburger D. The Strain of Being Chronically Ill. *Chronicity*. Springer. 2021. p. 21–24.

[33] Stephenson PS (2004). Understanding denial. Oncol Nurs Forum..

[34] Zhang Y, van Netten JJ, Baba M (2021). Diabetes-related foot disease in Australia: a systematic review of the prevalence and incidence of risk factors, disease and amputation in Australian populations. J Foot Ankle Res.

[35] Wellbeing SA. Lower limb amputations in South Australia. Government of South Australia.https://www.sahealth.sa.gov.au/wps/wcm/connect/public+content/sa+health+internet/about+us/wellbeing+sa/wellbeing+sa. Accessed 30 Feb 2023.

[36] Haleem A, Javaid M, Singh RP, Suman R (2021). Telemedicine for healthcare: capabilities, features, barriers, and applications. Sens Int.

[37] Nolan-Isles D, Macniven R, Hunter K (2021). Enablers and barriers to accessing healthcare services for Aboriginal people in New South Wales, Australia. Int J Environ Res Public Health.

[38] Kelly Haskard Zolnierek P, Robin DiMatteo P (2009). Physician Communication and Patient Adherence to Treatment. Med Care..

[39] Manzaneque NN, Pérez-Arechaederra D, Montalbán JMC (2019). Side effects and practices to improve management of type 2 diabetes mellitus from the viewpoint of patient experience and health care management. A narrative review. Endocrinol Diabetes Nutr (English ed)..

[40] Kwame A, Petrucka PM (2021). A literature-based study of patient-centered care and communication in nurse-patient interactions: barriers, facilitators, and the way forward. BMC Nurs.

[41] Litterbach E, Holmes-Truscott E, Pouwer F, Speight J, Hendrieckx C (2020). 'I wish my health professionals understood that it's not just all about your HbA1c!'. Qualitative responses from the second Diabetes MILES–Australia (MILES-2) study. Diabet Med..

[42] Bombard Y, Baker GR, Orlando E (2018). Engaging patients to improve quality of care: a systematic review. Implementation Sci.

